# Evidence for 2-Methoxyestradiol-Mediated Inhibition of Receptor Tyrosine Kinase RON in the Management of Prostate Cancer

**DOI:** 10.3390/ijms22041852

**Published:** 2021-02-12

**Authors:** Izhar Singh Batth, Shih-Bo Huang, Michelle Villarreal, Jingjing Gong, Divya Chakravarthy, Brian Keppler, Sridharan Jayamohan, Pawel Osmulski, Jianping Xie, Paul Rivas, Roble Bedolla, Michael A. Liss, I-Tien Yeh, Robert Reddick, Hiroshi Miyamoto, Rita Ghosh, Addanki P. Kumar

**Affiliations:** 1 Department of Molecular Medicine, University of Texas Health, San Antonio, TX 78229, USA; ibatth@gmail.com (I.S.B.); HuangS5@livemail.uthscsa.edu (S.-B.H.); villarreral@uthscsa.edu (M.V.); jgong@nanostring.com (J.G.); divyasc@gmail.com (D.C.); BKeppler@metabolon.com (B.K.); jayamohan@uthscsa.edu (S.J.); Osmulski@uthscsa.edu (P.O.); xiejianping2012@gmail.com (J.X.); rivasp@uthscsa.edu (P.R.); BEDOLLAR@uthscsa.edu (R.B.); 2 Urology, University of Texas Health, San Antonio, TX 78229, USA; Liss@uthscsa.edu (M.A.L.); ghoshr@uthscsa.edu (R.G.); 3 Mays Cancer Center, San Antonio, TX 78229, USA; 4 Pathology, University of Texas Health, San Antonio, TX 78229, USA; YEHI@uthscsa.edu (I.-T.Y.); REDDICK@uthscsa.edu (R.R.); 5 Department of Pathology and Laboratory Medicine, University of Rochester Medical Center, Rochester, NY 14642, USA; Hiroshi_Miyamoto@urmc.rochester.edu; 6 South Texas Veterans Health Care System, San Antonio, TX 78229, USA

**Keywords:** castration-resistant prostate cancer, receptor tyrosine kinase, RON, atomic force microscopy, 2-methoxyestradiol, epithelial mesenchymal transition, mechanical properties, bile acids, prostate cancer disparities

## Abstract

2-Methoxyestradiol (2-ME_2_) possesses anti-tumorigenic activities in multiple tumor models with acceptable tolerability profile in humans. Incomplete understanding of the mechanism has hindered its development as an anti-tumorigenic compound. We have identified for the first-time macrophage stimulatory protein 1 receptor (MST1R) as a potential target of 2-ME_2_ in prostate cancer cells. Human tissue validation studies show that MST1R (a.k.a RON) protein levels are significantly elevated in prostate cancer tissues compared to adjacent normal/benign glands. Serum levels of macrophage stimulatory protein (MSP), a ligand for RON, is not only associated with the risk of disease recurrence, but also significantly elevated in samples from African American patients. 2-ME_2_ treatment inhibited mechanical properties such as adhesion and elasticity that are associated with epithelial mesenchymal transition by downregulating mRNA expression and protein levels of MST1R in prostate cancer cell lines. Intervention with 2-ME_2_ significantly reduced tumor burden in mice. Notably, global metabolomic profiling studies identified significantly higher circulating levels of bile acids in castrated animals that were decreased with 2-ME_2_ intervention. In summary, findings presented in this manuscript identified MSP as a potential marker for predicting biochemical recurrence and suggest repurposing 2-ME_2_ to target RON signaling may be a potential therapeutic modality for prostate cancer.

## 1. Introduction

Prostate cancer (PCa) is the second leading cause of cancer in men in the US [1,2]. While localized PCa is not associated with a significant risk to mortality, a 5 year survival rate in those with metastatic disease is less than 30% [1]. One of the drivers of distant metastasis is the progression of hormone-naïve or hormone-sensitive PCa towards a hormone-independent form known as castration-resistant prostate cancer (CRPC). The androgen receptor (AR) is the primary driver of PCa growth and expansion. Its ability to initiate downstream signaling through ligand-independent mechanisms, such as interactions with receptor tyrosine kinases (RTKs), leads to the onset of CRPC [3,4].

Some RTKs have been shown to translocate to the nucleus and induce gene transcription, such as EGFR and c-MET [4,5,6]. Previously, we showed that the tyrosine kinase Recepteur d’origine nantais (RON), also known as macrophage stimulating-1 receptor (MST1R), was elevated in high grade (e.g., Gleason score ≥7) PCas, compared with lower grade (e.g., Gleason score ≤6) tumors [7]. RON is a cell membrane-associated receptor which is highly active in a variety of malignancies including prostate. Previous studies have found that RON is overexpressed in various types of cancers and indeed is a driver of metastasis and a potentiator of the aggressive phenotype [8,9,10,11,12,13,14,15]. Furthermore, prostate-specific overexpression or genetic suppression of RON demonstrate that RON is sufficient to promote PCa development [9,13,14]. More recent studies show that RON expression promotes prostate tumor growth following androgen deprivation [15]. Since androgen deprivation is a standard of care in patients with advanced PCa, this observation points to the importance of RON signaling in progression to CRPC. Previously we showed that RON contributed to modulating epithelial mesenchymal transition (EMT) and was also capable of activating AR signaling and its downstream targets (or “effectors”) such as prostate-specific antigen (PSA) and an anti-apoptotic gene c-FLIP [7]. These data suggest the therapeutic potential of RON/AR/c-FLIP axis to suppress PCa progression. Remarkably, published studies from our laboratory demonstrated that 2-Methoxyestradiol (2-ME_2_) (i) inhibited growth of PCa cells by downregulating Sp1-mediated transactivation of c-FLIP [16,17] and (ii) caused tumor regression with associated reduction in c-FLIP protein levels in transgenic adenocarcinoma of the mouse prostate (TRAMP) model [16,17]. It is noteworthy to mention that 2-ME_2_ was originally discovered as an anti-angiogenic agent that inhibits VEGF and HIF1α [18,19,20,21,22]. Of note, HIF1α was recently found to be a transcriptional activator of RON in breast cancer cells [23]. Studies also reported that RON is capable of inducing the secretion of angiogenic chemokines in PCa cell lines and promoting angiogenesis in prostate and breast cancer cells [15,24,25,26].

However, to the best of our knowledge, no published studies have examined the therapeutic ability of 2-ME_2_ to inhibit RON or RON-mediated signaling for PCa. Here, we investigated the potential of targeting RON using in vitro cell culture and in vivo preclinical model representing advanced PCa along with validation studies in human tumor specimens. Furthermore, we used unbiased global metabolomic profiling to determine the biochemical changes associated with castration in combination with 2-ME_2_ intervention in vivo.

## 2. Results

### 2.1. RON Levels Are Significantly Elevated in Prostate Adenocarcinoma

**Figure 1:**
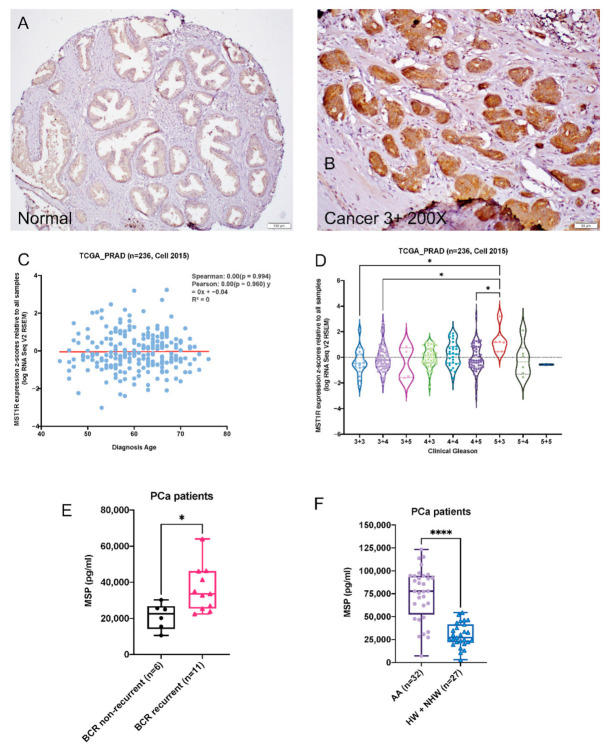
Alterations of RON in human prostate cancer cells and tumors. (**A**,**B**). Expression changes of RON in normal and human prostate tumors. (**C**). Scatter plot with regression straight line showing the correlation of MST1R mRNA expression in primary prostate tumor tissues and patients’ age in TCGA_PRAD dataset (*n* = 236) [27] determined by Spearman and Pearson correlation analysis. (**D**). Violin plot showing expression of MST1R in primary prostate tumor tissues categorized by Gleason status (TCGA_PRAD, *n* = 236) [27]. Each dot represents a single tumor tissue. Kruskal–Wallis Test was used for statistical analysis, * *p* < 0.05. Kruskal–Wallis Test was used for statistical analysis, * *p* < 0.05, **** *p* < 0.001. (**E**–**F**) Box plot shows MSP levels in sera of PCa patients with BCR (*n* = 11) and without BCR (*n* = 6; **E**) and AAs (*n* = 32), HWs and NHWs (*n* = 27; **F**). Statistical significance of the data was determined by Mann–Whitney test.

We used immunohistochemistry to stain for RON in a set of tissue microarray consisting of radical prostatectomy specimens. Analysis of these data revealed stronger RON staining (2+/3+) in carcinoma (*n* = 75) compared to adjacent normal prostate (*n* = 181; *p* = 0.002; Figure 1A,B). There was no significant association of RON expression with tumor grade or stage. Outcome analysis further revealed that strong RON staining (2+/3+) was associated with a higher risk of biochemical recurrence, compared with weak (0/1+) staining, although the difference was not statistically significant (*p* = 0.357; data not shown), possibly due to the small sample size. Overall, these results suggest a clinical relevance of RON in the pathogenesis of prostate cancer. These observations are consistent with our published data from a smaller number of samples (*n* = 28) [7]. In addition, although mRNA expression of MST1R (RON) showed no significant association with age of diagnosis, (Figure 1C) there was a significant increase in high grade (5 + 3) as compared with low grade (3 + 3 or 3 + 4) prostate tumors (Figure 1D). 

### 2.2. Serum MSP Level Is Associated with Biochemical Recurrence

Given that MSP is a secreted protein and RON ligand, we measured plasma levels using an ELISA-based assay (*n* = 59). We found that patients that experienced biochemical recurrence (*n* = 11) had significantly elevated MSP compared with patients that did not (*n* = 6; *p* = 0.0145; Figure 1E). These promising observations suggest an important role for RON signaling in disease progression. Further, these findings suggest that RON activity can be measured non-invasively and plasma levels of MSP can potentially be developed as a marker of aggressive cancer. Recently, we have reported elevated protein levels of RON in African American (AA) patients compared to Hispanic Whites (HW) and non-Hispanic Whites (NHW) combined [28]. Based on above observations, we measured levels of MSP in plasma samples from AAs and HWs and NHWs. Notably, MSP levels were significantly elevated in AAs (*n* = 32) relative to HWs and NHWs (*n* = 27) (*p* < 0.0001; Figure 1F). Remarkably, mRNA expression of MSTR1 was higher in a cell line derived from AA patient (MDA PCa 2b) compared with one derived from CA patient (LNCaP); there was an inverse relationship between AR and MSTR1 expression in these cell lines (Figure S1B). These data suggest that KLK3 (PSA) expression in AR^low^ AA cell line may be regulated through AR-independent mechanisms. All of these data taken together are consistent with a significant role for RON in PCa.

### 2.3. 2-ME_2_-Mediated Proliferation Inhibition Is Associated with Decreased Levels, Expression and Activity of RON in Advanced PCa Cell Lines

**Figure 2:**
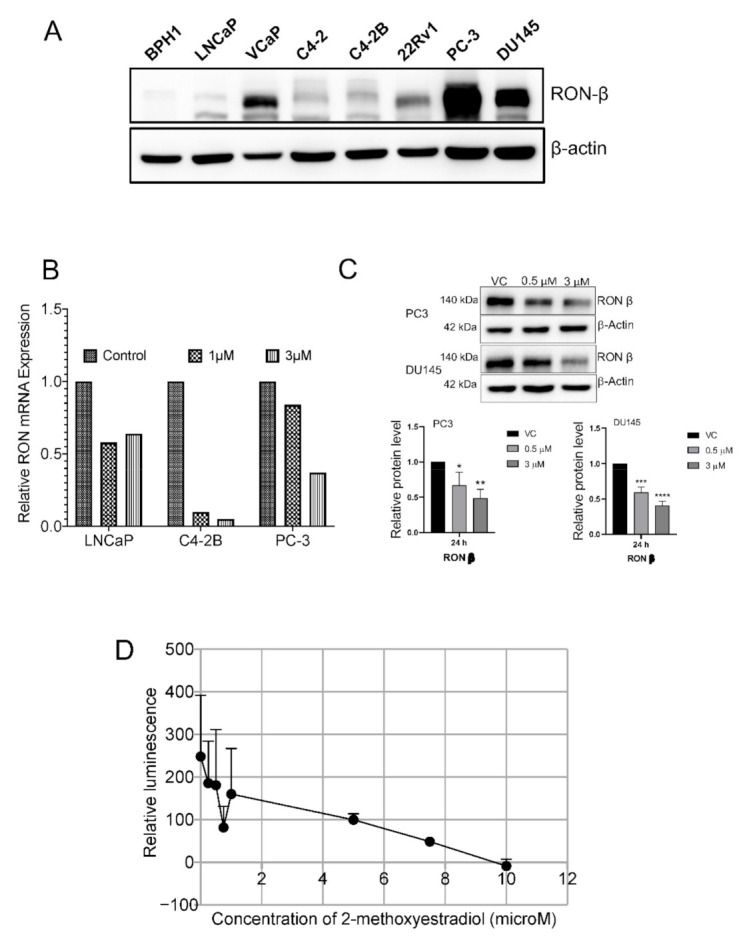
(**A**) Whole cell lysates from indicated cell lines was used to determine the protein levels of RON using immunoblot analysis. β-actin was used as the loading control. Blot shown are representative of two individual experiments. (**B**). RON mRNA levels following 24 h 2-ME_2_ treatment. Data presented is from 3 independent experiments. (**C**). Protein levels of RON following treatment with 2-ME_2_ (24 h) in PC-3 and DU145 cell lines using immunoblot analysis. β-actin was used as the loading control. A representative blot from three individual experiments is shown (**D**). Impact of 2-ME_2_ on RON kinase activity was determined using ADP-Glo^TM^ Kinase Assay (Promega Corporation, Madison, WI). The assay is based on monitoring the production of ADP concentration using luminescence which is directly proportional to kinase activity. Kinase reaction was performed essentially as per manufacturer’s protocol in the presence of increasing concentrations of 2-ME_2_. To test if 2-ME_2_ interferes with assay components, kinase reaction was performed excluding RON kinase and considered as background. Kinase activity is expressed as relative to kinase activity in the absence of 2-ME_2_. Data presented is an average of triplicate measurements.

Previously, we and others have reported the antitumorigenic activity for endogenous estrogenic metabolite namely, 2-methoxyestradiol (2-ME_2_) in multiple tumor models including prostate [16,17,18,19,20,21]. Specifically, we showed that intervention with 2-ME_2_ inhibits growth of PCa cells in vitro and tumor progression in vivo [16,17]. 2-ME_2_ treatment resulted in induction of apoptosis with associated decrease in antiapoptotic protein c-FLIP [16]. Furthermore, in recent studies, we found that RON transcriptionally regulates c-FLIP [7]. In order to examine the effect of 2-ME_2_ on RON, we examined RON protein levels in a panel of human PCa cells. Benign prostate epithelial cells (BPH-1) showed negligible RON protein. Androgen-responsive androgen receptor (AR) positive, LNCaP, C4-2 and C4-2B cells had lower levels followed by castration resistant AR v7 expressing 22Rv1 cells compared to AR overexpressing hormone refractory VCaP cells. In contrast, AR-negative PC-3 and DU145 cells showed the highest levels of RON protein (Figure 2A). Next, we examined the effect of 2-ME_2_ on mRNA expression and protein levels of RON. 2-ME_2_ treatment inhibited RON expression in all the cell lines tested (Figure 2B). 2-ME_2_ decreased RON protein levels in PC-3 and DU145 cells, with the highest level of RON (Figure 2C). Notably, 2-ME_2_ inhibited RON kinase activity by more than 50% at 3 μM in addition to decreasing protein levels at the same concentration (Figure 2D).

### 2.4. 2-ME_2_ Affects Mechanical Properties of PCa Cells

**Figure 3:**
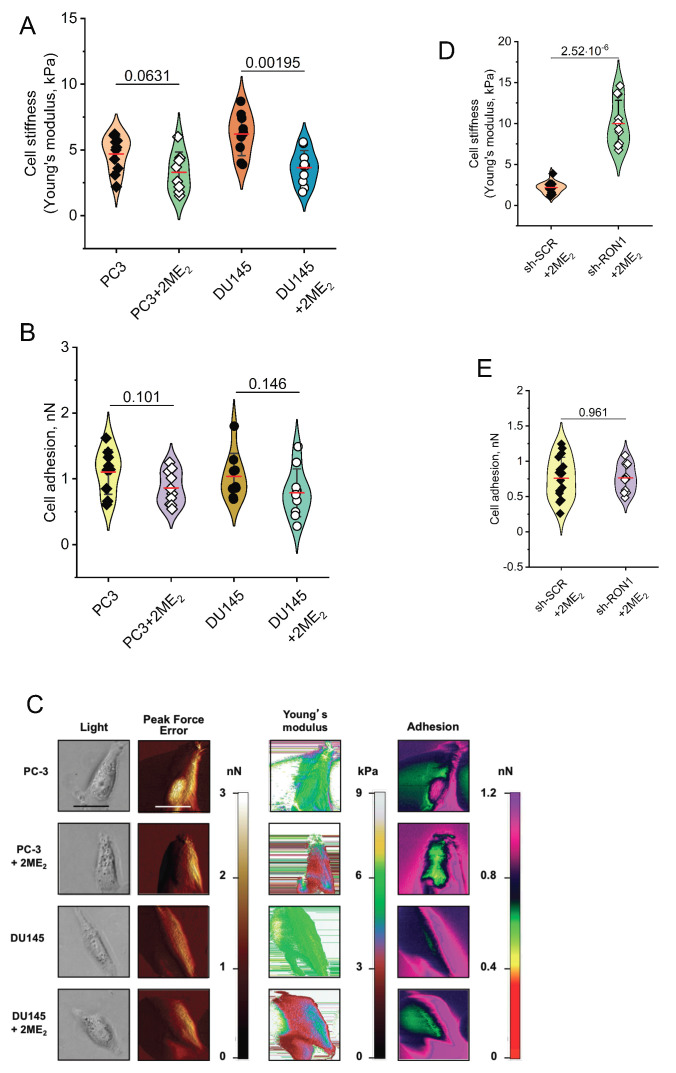
2-ME2 affects cell elasticity and adhesion of PCa cells (**A**–**E**). PC-3 and DU145 cells treated with 2-ME2 for 6 h at 3 μM. Increased stiffness of treated cells is reflected by a decrease of Young’s modulus (**A**). Treated cells are more adhesive (**B**). Each point represents an individual cell from repeated experiments. kPa = kiloPascals; pN = picoNewtons. (**C**) A panel of images obtained with the Peak Force QNM AFM shows distinct nanomechanical properties of individual PC-3 and DU145 control or 2-ME2-treated cells. Each row displays four different properties of a single cell with or without 2-ME2 treatment. Each column shows individual property of the cell collected in four channels: light microscopy image, peak force error (edge detection and fine topographical details), cell elasticity (Young’s modulus, kPa) and cell adhesion (nN). Peak Force Error images were used to determine the cell boundary collected in elasticity and adhesion channels. All images (except light microscopy) are false colored. The Peak Force Error scale shows smaller to taller objects progressing from dark brown to white color. The Young’s modulus (elasticity) scale shows softer objects as black and brown (lower modulus) and more rigid as green and yellow (higher modulus). The adhesion scale shows less adhesive objects as yellow and green (less force needed to separate an AFM tip from a cell) and stickier objects as dark blue and pink (more force needed). The black and white scale bars represent 40 and 20 μm, respectively (**D**,**E**). Mechanical properties of PC-3 cells stably silenced for RON in the presence and absence of 2-ME2 at 3 μM (6 h). Decreased stiffness is reflected by an increase of Young’s modulus (**D**). Adhesion of cells does not respond to silencing for RON and 2-ME2-treatement (**E**). Each point represents an individual cell.

Previously we showed that RON silencing changed mechanical properties and decreased markers associated with EMT [7]. Here we tested whether 2-ME_2_-mediated decreased levels of RON could impact the mechanical properties of PCa cells using atomic force microscopy (AFM) [7,29]. We found that treatment with 3 µM 2-ME_2_ for 6 h led to a marked increase in stiffness and a decrease in adhesion of both PC-3 and DU145 cells (Figure 3A–C). In order to determine a role for RON in mediating 2-ME_2_ effects on mechanical properties associated with EMT, we conducted similar experiments using RON silenced PC-3 cells. PC-3 shRON cells treated with 2-ME_2_ caused a decrease in cellular stiffness without impacting cell-cell adhesion compared with shNTC cells, suggesting that 2-ME_2_ mediates changes in mechanical properties through RON in these cells (Figure 3D,E).

**Figure 4:**
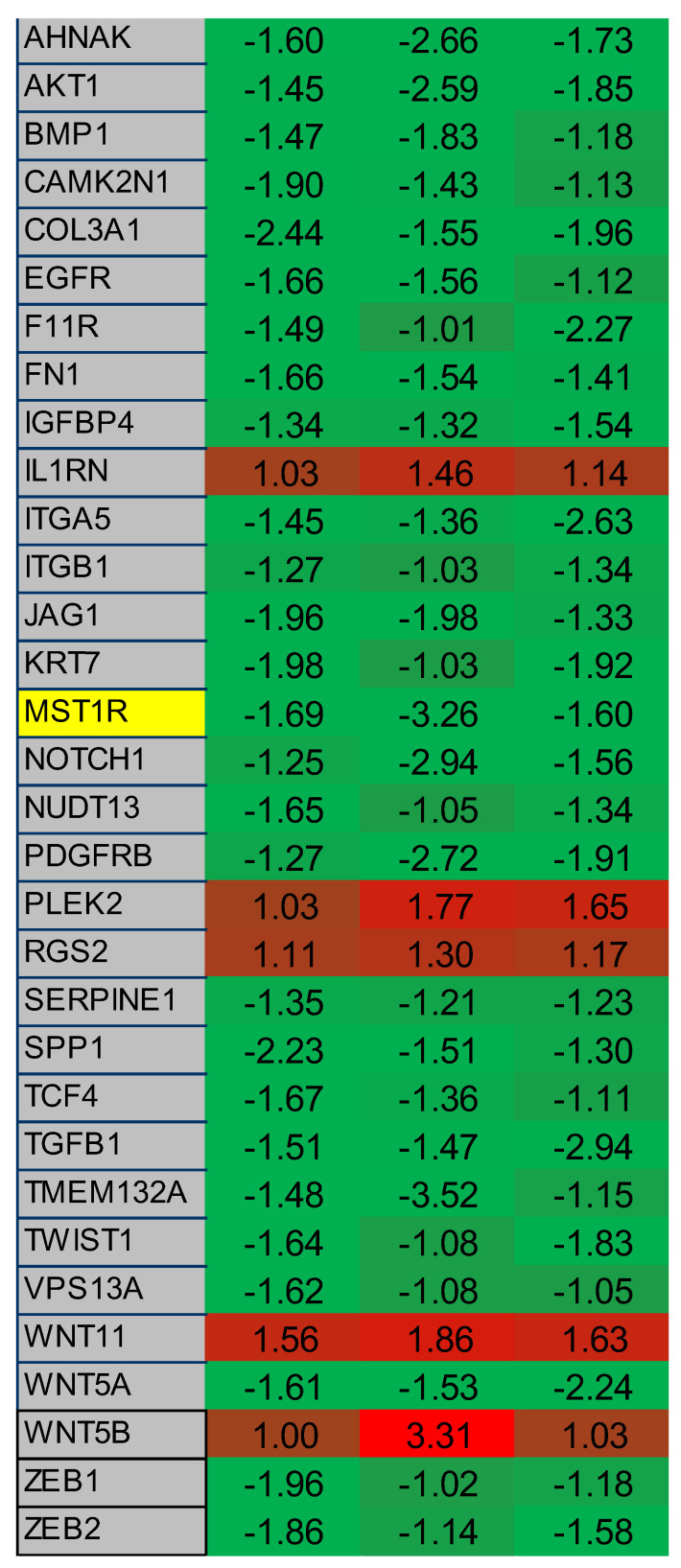
2-ME_2_ treatment in PC-3 cells affects multiple genes associated with EMT. A 96-well qPCR array of genes associated with EMT was screened using cDNA derived from control or 24 h 2-ME_2_ treated (3 µM) PC-3 cells. Each column represents a single experiment. Table shows fold change in gene expression relative to β-actin with consistent results across three experiments. RON (listed as MST1R) is highlighted in yellow. A heatmap was created based on relative overexpression (red) and under expression (green). Green indicates upregulated gene expression from 0 to +4 and red indicates downregulated gene expression from 0 to −4.

The results presented so far suggest that 2-ME_2_ is a modulator of RON and affects mechanical properties such as adhesion and stiffness that are indicative of EMT. This is in addition to its previously known roles as an inducer of apoptosis and inhibitor of angiogenesis [16,30,31,32,33,34,35]. It is well established that EMT plays an important role in progression to advanced cancer including prostate [36,37,38,39,40,41]. Multiple molecular pathways, including growth factor, cytokine, inflammatory and cell signaling, have been shown to regulate EMT process [36,37,38,39,40,41]. Based on these observations showing potential role for 2-ME_2_ in modulating EMT, we examined a commercially available EMT-pathway specific qPCR array containing genes including RON in PC-3 cells. Consistent with data presented above showing the ability of 2-ME_2_ to reduce levels and expression of RON, analysis of EMT-pathway-specific array data revealed reduced expression of RON. Remarkably, master regulators of EMT namely TWIST and ZEB1 were downregulated in response to 2-ME_2_ treatment (Figure 4 and Figure S1C). These data suggest that besides inducing apoptosis and blocking angiogenesis, 2-ME_2_ modulates EMT by changing the mechanical properties of PCa cells.

### 2.5. Heterogeneous Response to Nano-Coated 2-ME_2_ Intervention for 10 Weeks on Castrate Resistant Prostate Tumor Development

**Figure 5:**
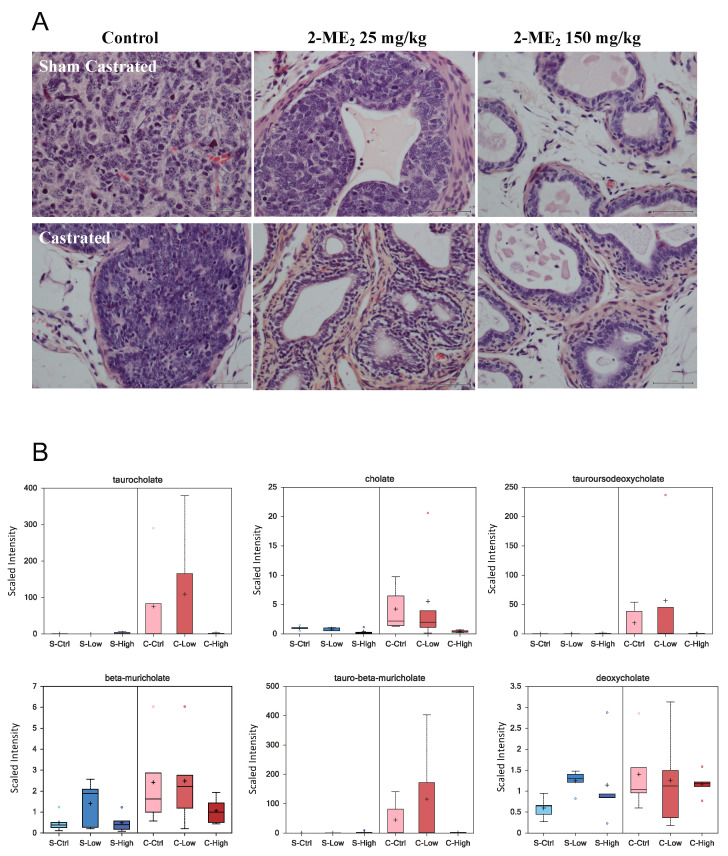
(**A**). Sham castrated or castrated TRAMP mice received water control (*n* = 10 each for sham or castrated group) or 25 or 150 mg/kg 2-ME_2_ (*n* = 10 each for sham or castrated group per respective dose). The experiment was terminated following 10 week-intervention and prostate tumor, or tissue was collected for histopathological evaluation. A representative image of H&E staining is shown. (**B**) Box plots showing levels of indicated bile acids in serum from castrated or sham-castrated mice (*n* = 5 per each group).

Although 2-ME_2_ has previously been tested in vivo in various tumor models including our own studies in PCa, its ability to inhibit CRPC has not been tested [16,17]. Therefore, we tested the ability of nano-coated 2-ME_2_ with enhanced bioavailability to prevent progression to CRPC in transgenic adenocarcinoma of mouse prostate model (TRAMP). We selected this mouse model due to its ability to develop the disease in a reasonable time frame including progression to castration resistance and the histological similarities to the human PCa [42,43]. Histopathological evaluation of the tumors at the termination of the experiment revealed that prostate tumors from sham-castrated control group animals exhibited pathological features consistent with poorly differentiated to well-differentiated adenocarcinoma, whereas tumors from the castrated control group animals showed both poorly differentiated and high-grade PIN lesions. On the other hand, the observed pathological features ranged from high grade PIN, well to moderately differentiated adenocarcinoma in response to intervention with 2-ME_2_ in both castrated and sham-castrated groups. Analysis of these data indicated statistically significant 2.25-fold (*p* = 0.0112) increased risk of prostate cancer development without 2-ME_2_ under sham-castrated conditions. Intervention with 2-ME_2_ for 10-weeks with castration provided no additional benefit in this cohort of castrated mice (*p* = 0.81). Previously we reported oral administration of 50 mg/kg 2-ME_2_ suppressed prostate tumor growth using TRAMP model [16]. In contrast, we were able to achieve tumor growth suppression using 25 mg/kg nano-coated 2-ME_2_. Although we have not conducted bioavailability studies, our results suggest improved bioavailability may account for this observation [44]. Following castration, although several animals exhibited pathological features consistent with normal or HGPIN lesions, the observed differences did not reach statistical significance possibly be due to small sample size or intervention duration. Our results are encouraging, suggesting future test of longer duration of combination therapy to determine efficacy. A representative histopathological evaluation of the prostate is presented in Figure 5A. Pathological analysis of prostate tumors from individual animals is presented in Figure S2.

The above data suggest that 2-ME_2_-intervention can have histopathological response under sham-castrate conditions. However, the precise biochemical alterations and/or molecular events associated with progression to castrate resistance is undefined. Defining such biochemical alterations following therapy would pave the way to identify novel markers of response to treatment. Therefore, we employed mass spectrometry-based profiling of the metabolome to identify changes in biochemicals under these experimental conditions. We identified a total of 54 biochemicals of which 16 increased and 38 decreased in castrated animals compared with sham-castrated animals. Treatment of sham-castrated animals with low and high 2-ME_2_ altered 91 and 145 biochemicals, respectively. On the other hand, treatment of castrated animals modulated 89 and 106 biochemicals. Experimental design and the data showing changes in biochemicals (increase and decrease) is presented in Figure S3. Analysis of these data also identified alteration of 60 biochemicals associated with castration effect, 149 with treatment and 70 interactions between castration and treatment effects. Castration affected metabolites involved in a variety of metabolomic pathways including lipid, oxidative stress, energetics and bile acids. As shown in Figure 5B, we found castration-induced increased levels of cholate, taurocholate, tauroursodeoxycholate, tauro-beta-muricholate, beta-muricholate and deoxycholate. Interestingly, 2-ME_2_ intervention attenuated the observed changes either dose dependently or only at higher dose. Although the potential implication of these changes is not entirely clear, published reports show elevated levels of bile acids and their metabolites in prostate cancer patients with T4 disease [45] and also following androgen deprivation therapy for 3 months [46].

## 3. Discussion

In our translational research study, we describe the association of MSP in recurrent PCa and the ability of 2-ME_2_ to inhibit this pathway. Screening for PCa is declining due to several factors and is unfortunately leading to an increasing incidence of aggressive cancer or metastatic disease at diagnosis [47]. Therefore, biomarkers are needed to focus on the identification of potentially aggressive tumors at an early stage of PCa development to maximize therapeutic efficacy [3].

RTKs have primarily been understood as extra-nuclear, cell surface signaling initiators and conveyers that do not have direct interaction with the cell’s transcription machinery [48,49]. However, there are notable exceptions to this rule such as EGFR’s ability to translocate to the nucleus as well as other organelles in cancer cells [50,51,52]. c-MET and RON kinase have both been previously mentioned as having nuclear activity [53,54,55]. We and others have demonstrated that RON plays a role in progression to advanced PCa [7,9,15]. Therefore, targeting RON as part of a PCa treatment regimen may be advantageous. However, currently available clinical compounds capable of inhibiting RON are effective against multiple RTKs, have greater affinity for other kinases [55]. Therefore, there is an unmet need to identify and develop compounds that can inhibit RON for therapeutic benefit.

Previously we and others demonstrated that 2-ME_2_, a non-toxic anti-tumorigenic compound exerts growth inhibitory effects both in vitro and in vivo in multiple tumor models including prostate [16,17,18,19,20,21,30,31,32,33,34,35]. 2-ME_2_ is primarily known as a pro-apoptotic and anti-angiogenic compound [16,17,18,19,20,21,30,31,32,33,34,35]. Studies from our laboratory demonstrated that inhibition of c-FLIP activation using 2-ME_2_ reduced prostate tumor development in a preclinical animal model [16]. Although 2-ME_2_ has been reported to inhibit cancer cell growth through induction of apoptosis and inhibition of angiogenesis, the precise molecular mechanism is unclear. In this study, we provided evidence regarding the ability of 2-ME_2_ to reduce levels and expression of RON in PCa cell lines. In addition to the known traditional pro-apoptotic and anti-angiogenic role, results presented in this manuscript suggest a role for 2-ME_2_ in modulating EMT in part through RON as evidenced by change in mechanical properties through AFM and changes in gene expression associated with EMT. Specifically, 2-ME_2_ mimicked RON inhibition in terms of changes in mechanical properties [7]. It is possible that 2-ME_2_ could regulate RON through an intermediate factor. For example, 2-ME_2_ is known to be anti-angiogenic and can target HIF-1α [19]. RON has been shown to be a downstream target of HIF1α in other cancers and as such, is a potential downstream or direct target of 2-ME_2_ [18,23]_._ These publications, as well as our own data point towards a regulatory function of 2-ME_2_ over RON.

We also discovered elevated plasma levels of MSP in patients with a recurrence of their PCa. Remarkably, plasma levels of MSP were higher in AA patients relative to NW plus NHWs, and RON expression was higher in a cell line derived from AA patient (MDA PCa 2b) compared to LNCaP (derived from a Caucasian patient). We hypothesize that MSP may allow tumors to grow in a non-androgen dependent pathway. Furthermore, serum from AA patients showed significantly elevated levels of MSP compared to PCa is the most commonly diagnosed cancer (30%) in AAs and contributes to 15% of cancer related deaths [1,28,56,57,58]. While 1 of 7 black men develop and 1 of 25 die from PCa, these statistics are distinctly different from white men of European descent with PCa diagnosis (1 in 9 for incidence and in 1 of 45 for death). According to the American Cancer Society, approximately 29,570 black men are expected to be diagnosed with PCa and about 5350 are to die of the disease in 2019. Black men are at 1.3 times higher risk with highest death rate for PCa compared to whites [56,57,58]. Published studies also show increased rates of progression to recurrence among men of African origin undergoing surgery [56,57,58]. Therefore, we believe that repurposing 2-ME_2_ to downregulate RON could be developed as an approach for management of castrate resistant prostate cancer especially in blacks and other tumors that exhibit elevated activation of RON signaling.

It is possible that the observed reduced incidence of aggressive cancer in response to 2-ME_2_ in sham-castrated mice could be due to slower rate of tumor progression as we performed pathological evaluation at the time of termination of the experiment. Currently available therapies for prostate cancer mostly deal with androgen blockade or chemical castration, many of which have multiple side effects such as impotence, musculoskeletal pain, hot flashes, anemia, diarrhea, anxiety and hypertension and metabolic affects, in addition to being a great financial burden for the patients [3,59,60]. Data presented in this manuscript also implicate a potential role for bile acids in progression to castration resistance providing a framework for therapeutic targeting bile acid metabolism. For example, choline and intermediates were increased in castration-resistant conditions compared to sham castration and decreased upon treatment with 2-ME_2_. Bile acids produced from liver modulate numerous metabolic pathways including nuclear receptor signaling, oxidative stress and tumor promotion [61,62,63]. Although the reasons for the observed castration-induced changes in bile acids is unclear, given that bile acids can be modified by gut microflora, it is tempting to speculate a role for alterations in gut microflora following castration. Further investigations are needed to test whether alterations in bile acids could activate RON signaling under castration conditions. A phase III trial using Cabozantinib, a dual inhibitor of c-MET/VEGFR2 has not shown survival benefit in men with CRPC [64]. A recent study shows that Cabozantinib in combination with enzalutamide was more efficacious than single agent in LNCaP xenograft model [65]. Data presented in this manuscript suggest potential for repurposing 2-ME_2_ a well-studied molecule with good safety profile, a pro-apoptotic and anti-angiogenic agent to downregulate mRNA expression of number of genes involved in tumor metastasis including RON.

## 4. Materials and Methods

### 4.1. Cell Lines

LNCaP, PC-3, C4-2B and DU145 were obtained from American Type Culture Collection (ATCC, Rockville, MD, USA) and cultured as described by us previously [66,67,68]. BPH-1, LNCaP, and DU145 cell lines were grown in RPMI media supplemented with 10% FBS and 1% antibiotics. C4-2B cells were grown in T-Media supplemented with 5% FBS and 1% antibiotics. PC-3 cells were grown in F12K media supplemented with 10% FBS and 1% antibiotics. All cell lines were maintained in a T75 cell culture flask in a 37 °C incubator with 5% CO_2_.

### 4.2. Animal Study

The 8–10-week-old sham-castrated and castrated TRAMP mice obtained from Jackson Laboratories (Maine, FL, USA) were randomized into six groups of 10 animals each (three groups for age-matched sham-castrated and three groups of castrated). Each of the sham-castrated and castrated groups of mice received 0, 25 and 150 mg/kg body weight 2-ME_2_ in drinking water for 10 weeks. These doses were used based on our previously published studies [16,17]. Institutional Animal Care and Use Committee approved the protocol. 2-ME_2_ was obtained from Entramed, Inc.

### 4.3. RNA and qPCR

Total RNA was extracted using TRIZOL and used to generate cDNA for gene expression experiments [66,67,68,69]. Expression of target gene mRNA transcripts was determined by Realtime PCR with gene-specific primers and SYBR-green PCR mix (Life Technologies). SA Biosciences RT^2^ 96-well human EMT-associated genes qPCR array was used to determine whether any such genes are affected with 2-ME_2_ treatment in PC3 cells. All qPCR data was acquired using the Abi 7300 or Bio-Rad CFX96 systems. The following primers were used for the listed genes to determine mRNA changes.

β-Actin Forward: 5′-GGCACCCAGCACAATGAAGATCAA-3′

β-Actin Reverse: 5′-TAGAAGCATTTGCGGTGGACGATG-3′

RON Forward: 5′-AGCCCACGCTCAGTGTCTAT-3′

RON Reverse: 5′-GGGCACTAGGATCATCTGTCA-3′

### 4.4. RNA Interference and Plasmid Transfection

In transient silencing experiments, logarithmically growing DU145 and PC-3 cells were transfected with 25 and 50 nM ON-TARGETplus Human MST1R siRNA smart pool (Dharmacon), respectively, using Lipofectamine 2000 according to manufacturer’s recommendation. 48–72 h after transfection, levels and expression of RON were analyzed by western blot and qPCR, respectively. Stable RON knockdowns in PC3 and DU145 cells were achieved using Qiagen MST1R SureSilencing shRNA Plasmid (KH07170) with puromycin selection marker. Mixed populations of cells stably transfected with the single sh-Scrambled plasmid construct or one of the four provided sh-RON constructs were generated and used for experiments.

### 4.5. Western Blotting

Whole cell extracts were prepared using 2X SDS lysis buffer containing protease and phosphatase inhibitors. Lysates were placed in boiling water for 10 min and used for fractionation on 8% SDS gels. Proteins from the gel were transferred on to nitrocellulose membrane and probed with the appropriate antibodies. The following antibodies and their dilutions were used: Santa Cruz Biotechnology RON (sc-322, 1:2000), Anti-Mouse (sc-2005, 1:2000). Sigma Aldrich—β-Actin (A5316, 1:5000), Anti-Rabbit (A6154, 1:2000).

### 4.6. Immunohistochemistry

Tissue microarray (TMA) consisting of prostate specimens from radical prostatectomy performed at the University of Rochester Medical Center was constructed upon appropriate approval from the institutional review board. H&E-stained TMAs were manually scored to determine the levels of RON for tumor grade as previously described [66]. Stains were manually scored by a single pathologist (H.M.) who was blinded to sample identity. IHC scores were derived by multiplying the percentage of immunoreactive cells (0% = 0; 1–10% = 1; 11–50% = 2; 51–80% = 3; 81–100% = 4) by staining intensity (negative = 0; weak = 1; moderate = 2; strong = 3) (final score: 0–1 = negative (0); 2–4 = weakly positive (1+); 6–8 = moderately positive (2+); 9–12 = strongly positive (3+).

Rabbit polyclonal antibodies specific for RON was from Santa Cruz Biotechnology (Carpenteria, CA, USA), rabbit polyclonal AR antibody was from Thermo Scientific (Fremont, CA, USA) were used in IHC. Staining was performed using standard IHC methods, including the use of appropriate negative and positive controls. Briefly, the tissues were de-paraffinized and rehydrated, then they went through heat antigen retrieval (citrate) followed by hydrogen peroxide and protein blocking. The tissues were incubated one hour at room temperature with the antibodies. The ancillary system used was rabbit HRP polymer and DAB chromogen, and then counterstained with hematoxylin (DAKO North America Inc. Carpentaria, CA, USA).

### 4.7. Atomic Force Microscopy (AFM)

AFM analysis was performed essentially as described before [7,68,69,70]. Adherent DU145, PC-3 and PC-3-shRON cells immersed in a culture medium in the presence and absence of 3 µM 2-ME_2_ were directly scanned with AFM in 55 mm uncoated Petri dishes without any additional processing or immobilization. Cells from a single dish were imaged for up to 90 min without morphological signs indicating loss of their viability. Cells were scanned with a Nanoscope Catalyst (Bruker) AFM mounted on a Nikon Ti inverted epifluorescent microscope using the PeakForce Quantitative Nanomechanical Mapping (PF-QNM) mode (Bruker). Before AFM imaging, a light microscopic image was recorded for each cell. Scanning of a single cell took from 12 to 15 min. Electronic resolution of 30 × 30 to 50 × 50 μm square images varied from 64 × 64 to 256 × 256 pixels (×, number of points per line by y, number of lines). SCANASYST-AIR (Bruker) probes were used for imaging. The spring constant of the nominal value 0.02 N/m was determined for each probe with the thermal tuning. To avoid possible imaging artifacts arising from dense indentation of a cell surface with a tip we applied a relatively large peak force amplitude between 1000 and 1500 nm and slow scan rates up to 0.3 Hz. To keep cell membrane integrity the maximum allowed indentation was lower than 200 nm. To determine cell boundaries, a cell shape and nanotopography was collected in height and peak force error channels, respectively. In parallel, the nanomechanical data consisting of cell stiffness and adhesion were captured in two additional separate channels. Individual force plots for each pixel were gathered in peak force capture (pfc) files enabling complete adjustment of background and application of different stiffness models and validation of calculation. Nanomechanical parameters were calculated with Nanoscope Analysis software v. 4.1 using the retrace images. Calculation of the elastic modulus factorized the assumed high heterogeneity of cell surface. Additionally, we included adhesion forces in all the analysis. Calculations were performed based on the Sneddon model that approximates the mechanics of conical tip interactions with an object. A mode value of stiffness and adhesion for each cell was extracted from corresponding distribution histograms and applied in all the downstream statistical evaluations.

### 4.8. Metabolomic Profiling

The untargeted metabolomic profiling platform employed for this analysis was accomplished by Metabolon and combined three independent platforms: ultrahigh performance liquid chromatography/tandem mass spectrometry (UPLC/MS/MS) optimized for basic species, UPLC/MS/MS optimized for acidic species, and gas chromatography/mass spectrometry (GC/MS). Samples were processed essentially as described previously [71,72,73,74,75]

Using an automated liquid handler (Hamilton LabStar, Salt Lake City, UT, USA), protein was precipitated from the samples with methanol that contained four standards to report on extraction efficiency. The resulting supernatant was split into equal aliquots for analysis on the three platforms. Aliquots, dried under nitrogen and vacuum-desiccated, were subsequently either reconstituted in 50 μL 0.1% formic acid in water (acidic conditions) or in 50 μL 6.5 mM ammonium bicarbonate in water, pH 8 (basic conditions), for the two UPLC/MS/MS analyses or derivatized to a final volume of 50 μL for GC/MS analysis using equal parts bistrimethyl-silyl-trifluoroacetamide and solvent mixture acetonitrile:dichloromethane:cyclohexane (5:4:1) with 5% triethylamine at 60 °C for one hour. In addition, three types of controls were analyzed in concert with the experimental samples: samples generated from pooled experimental samples served as technical replicates throughout the data set, extracted water samples served as process blanks, and a cocktail of standards spiked into every analyzed sample allowed instrument performance monitoring. For UPLC/MS/MS analysis, aliquots were separated using a Waters Acquity UPLC (Waters, Millford, MA, USA) and analyzed using an LTQ mass spectrometer (Thermo Fisher Scientific, Inc., Waltham, MA, USA) which consisted of an electrospray ionization (ESI) source and linear ion-trap (LIT) mass analyzer. The MS instrument scanned 99–1000 *m/z* and alternated between MS and MS^2^ scans using dynamic exclusion with approximately 6 scans per second. Derivatized samples for GC/MS were separated on a 5% phenyldimethyl silicone column with helium as the carrier gas and a temperature ramp from 60 °C to 340 °C and then analyzed on a Thermo-Finnigan Trace DSQ MS (Thermo Fisher Scientific, Inc., Waltham, MA, USA) operated at unit mass resolving power with electron impact ionization and a 50–750 atomic mass unit scan range.

### 4.9. Metabolite Identification and Data Analysis

Metabolites were identified by automated comparison of the ion features in the experimental samples to a reference library of chemical standard entries that included retention time, molecular weight (*m/z*), preferred adducts, and in-source fragments as well as associated MS spectra and curated by visual inspection for quality control using software developed at Metabolon. Experimental samples and controls were randomized across a three-day platform run. To account for differences in biochemical levels that are attributed to differences in cell numbers between samples, the levels of each biochemical in a given sample were normalized to DNA concentration. To correct for instrument inter-day tuning differences without masking sample differences, a data normalization step was performed. Each compound was corrected in run-day blocks by registering the medians equal to one (after DNA normalization) and normalizing each data point proportionately. Any missing values were assumed to be below the limits of detection and for statistical analyses and data display purposes, these values were imputed with the compound minimum (minimum values imputation) after the data normalization step. Statistical analysis of log-transformed data was performed using “R” (http://cran.r-project.org/ (accessed on 11 February 2021)), which is a freely available, open-source software package. Welch’s t-tests were performed to compare data between experimental groups. Multiple comparisons were accounted for by estimating the false discovery rate (FDR) using *q*-values [76].

### 4.10. RON Kinase Activity

RON kinase activity was measured essentially as per manufacturers recommendation using the ADP-Glo^TM^ Assay and the RON Kinase Enzyme System (Promega Corporation, Madison, WI, USA). This luminescence-based assay measures the kinase activity by quantifying the amount of ADP produced following treatment with different concentrations of 2-ME_2_. The luminescence of the 96-well plate was recorded at an integration time of 0.5 s using the SpectraMax M5 machine (Molecular Devices, San Jose, CA, USA).

### 4.11. MSP in Serum Samples

The study was conducted according to the guidelines of the Declaration of Helsinki and approved by the Institutional Review Board (IRB) of University of Texas Health, San Antonio, TX. Serum samples used in the study were obtained from IRB approved (i) Circulating Tumor Cell study (HSC20130219H) that enrolls men initiating hormone deprivation therapy and (ii) men participated in active surveillance (HSC20150160H) study following diagnosis with low grade PCa. In both studies, men are followed for cancer recurrence through serial collection of blood samples. Total amount of MSP proteins in sera of PCa patients were measured according to manufacturer’s instructions using Microphage Stimulating Protein Human ELISA Kit (Abcam ab100612, Cambridge, UK). Absorbance at 450 nm was measured using a SpectraMax M5 plate reader (Molecular Devices).

### 4.12. Bioinformatic Analysis

Expression of MST1R in human primary prostate adenocarcinoma RNA-Seq RSEM expression data and clinical information of patients including age, Gleason score and tumor stage were extracted from The Cancer Genome Atlas (TCGA) [27,77] and downloaded from the cBioPortal website [78,79].

### 4.13. Statistical Analysis

Statistical significance of the associations between tumor grades, tumor Gleason score and expression of MST1R in human primary prostate tumor tissues (TCGA cohort) was determined by One-way analysis of variance (ANOVA) Kruskal–Wallis Test following D’Agostino-Pearson omnibus K2 normality test on GraphPad Prism 9 software. The correlation between patients’ ages and expression of MST1R in human primary prostate tumor tissues (TCGA cohort) was determined by Pearson and Spearman correlation analysis. All significance level was set at 0.05 (* *p* < 0.05, and *** *p* < 0.001).

## Data Availability

Data can be obtained from the corresponding author.

## Supplementary Materials

The following are available online at https://www.mdpi.com/1422-0067/22/4/1852/s1, Figure S1: **A**. Violin plot showing expression of MST1R in primary prostate tumor tissues classified by pathological stages extracted from TCGA_PRAD dataset (*n* = 486, PanCancer Atlas). Each dot represents a single tumor tissue. **B**. Expression of MST1R, AR and KLK3 in LNCaP and MDA PCa 2b were extracted from the Cancer Cell Line Encyclopedia (CCLE) data [80]. A heatmap was created based on relative overexpression (red) and under expression (green) using the Multi-Experiment Viewer (MeV 4.9) software. **C**. 2-ME2 treatment in PC-3 cells affects multiple genes associated with EMT. A 96-well qPCR array of genes associated with EMT was screened using cDNA derived from control or 24h 2-ME2 treated (3 µM) PC-3 cells. Each column represents a single experiment. Data shows fold change in gene expression relative to β-actin. A heatmap was created based on relative overexpression (red) and under expression (green). Figure S2: Pathological analysis of prostate tumors from individual animals from preclinical study. Figure S3: Experimental description of metabolomic analysis from serum samples.
