# Supplementary material for: Evidence for 2-Methoxyestradiol-Mediated Inhibition of Receptor Tyrosine Kinase RON in the Management of Prostate Cancer
Source: Int J Mol Sci. 2021 Feb 12;22(4):1852. doi: 10.3390/ijms22041852 (PMC7918140; doi:10.3390/ijms22041852)
Supplement: Supplementary file 1 [file ijms-22-01852-s001.pdf]

Supplementary data Figure 1

A

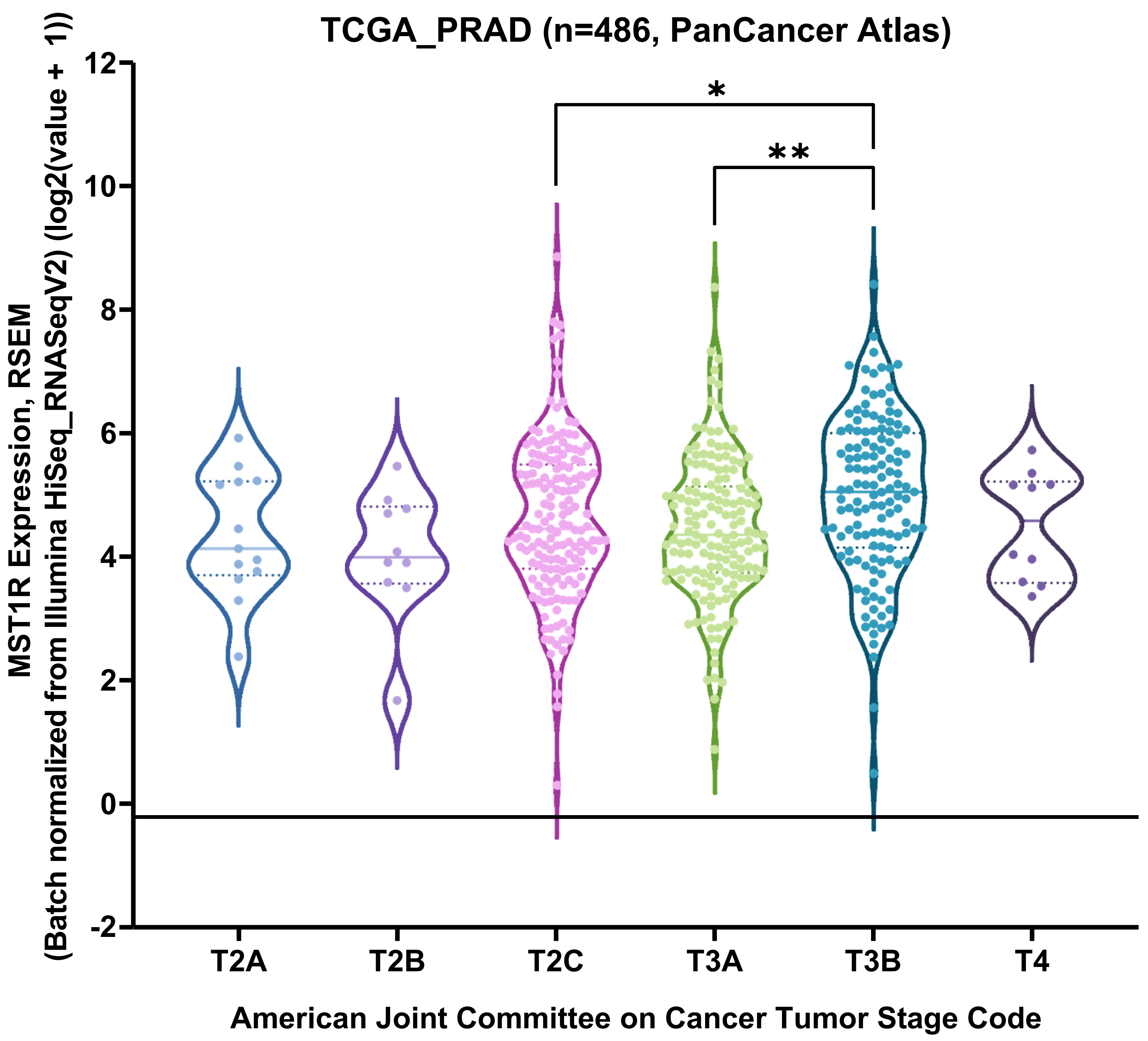

B

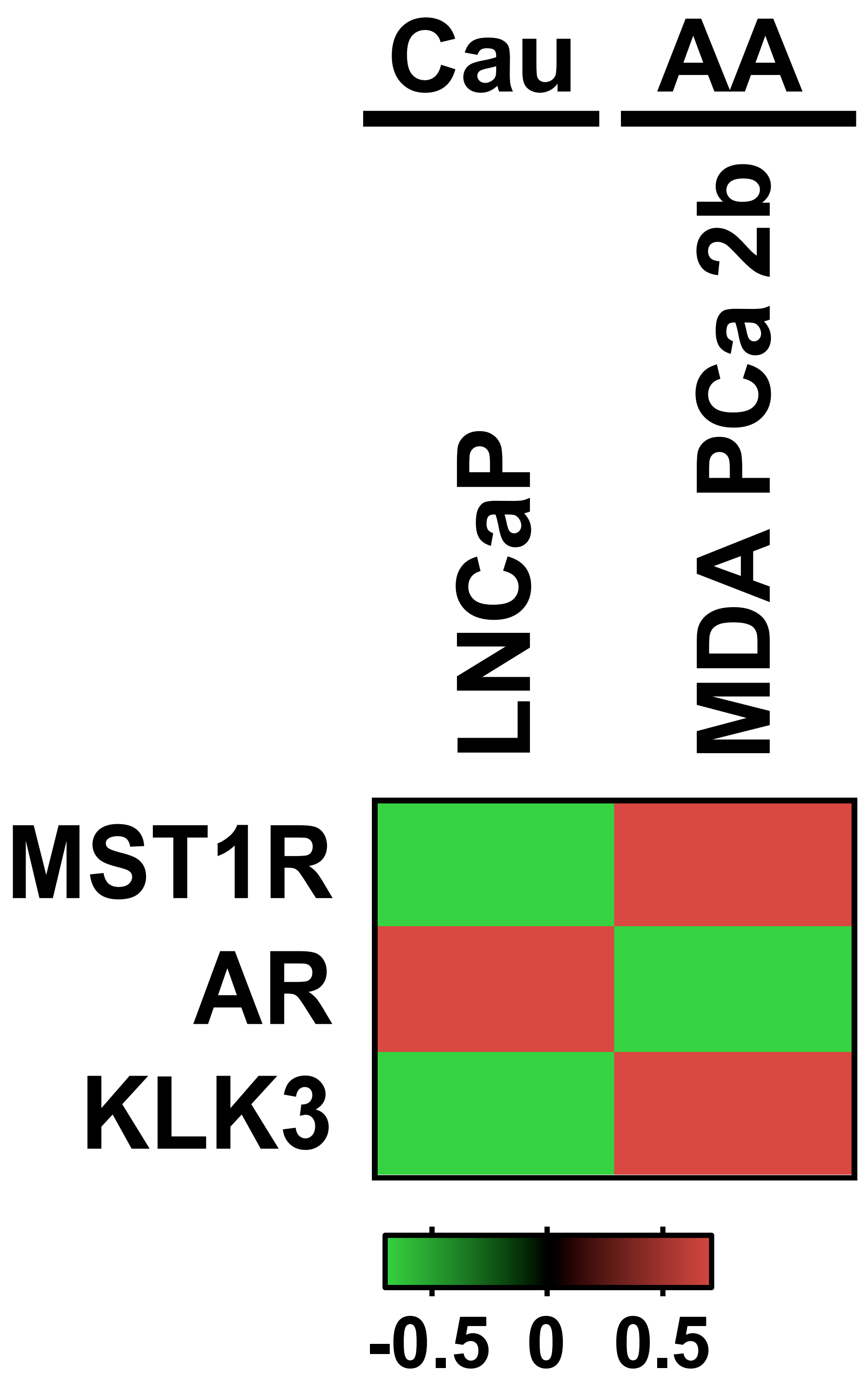

C

AHNAK	-1.60	-2.66	-1.73
AKT1	-1.45	-2.59	-1.85
BMP1	-1.47	-1.83	-1.18
BMP7	-1.41	1.94	-3.62
CALD1	-1.40	-1.18	1.11
CAMK2N1	-1.90	-1.43	-1.13
CAV2	-1.58	1.23	1.03
CDH1	-1.23	1.49	-1.67
CDH2	-1.82	1.50	-1.66
COL1A2	-1.96	4.88	-1.33
COL3A1	-2.44	-1.55	-1.96
COL5A2	-1.88	1.04	-1.60
CTNNB1	-1.20	1.11	-1.01
DSC2	-1.32	1.38	-1.06
DSP	-1.71	1.01	1.03
EGFR	-1.66	-1.56	-1.12
ERBB3	-1.53	-1.23	1.25
ESR1	1.19	1.12	-2.21
F11R	-1.49	-1.01	-2.27
FGFBP1	-1.55	1.60	-2.06
FN1	-1.66	-1.54	-1.41
FOXC2	1.12	-1.56	1.03
FZD7	-1.63	-1.07	1.15
GNG11	1.05	-1.14	-1.11
GSC	-1.32	1.44	-1.23
GSK3B	-1.00	-1.34	1.70
IGFBP4	-1.34	-1.32	-1.54
IL1RN	1.03	1.46	1.14
ILK	-1.02	1.15	-1.23
ITGA5	-1.45	-1.36	-2.63
ITGAV	-1.31	1.32	-1.35
ITGB1	-1.27	-1.03	-1.34
JAG1	-1.96	-1.98	-1.33
KRT14	1.19	1.01	-1.14
KRT19	-1.30	1.29	-1.55
KRT7	-1.98	-1.03	-1.92
MAP1B	-1.71	-1.33	1.05
MITF	-1.14	1.03	1.45
MMP2	-1.18	-1.18	1.32
MMP3	-1.58	-1.55	1.38
MMP9	1.02	1.86	1.99
MSN	-1.90	1.35	-1.99
MST1R	-1.69	-3.26	-1.60
NODAL	-1.45	-2.02	1.13
NOTCH1	-1.25	-2.94	-1.56
NUDT13	-1.65	-1.05	-1.34
OCLN	-1.20	1.45	1.32
PDGFRB	-1.27	-2.72	-1.91
PLEK2	1.03	1.77	1.65
PPPDE2	-1.72	1.26	-1.03
PTK2	-1.80	1.39	-2.47
PTP4A1	-1.30	1.19	-1.10
RAC1	-1.40	1.93	-1.04
RGS2	1.11	1.30	1.17
SERPINE1	-1.35	-1.21	-1.23
SIP1	-1.23	1.15	1.16
SMAD2	-1.55	1.29	1.05
SNAI1	-1.40	1.48	-1.77
SNAI2	-1.27	1.62	1.32
SNAI3	-1.09	-1.03	1.82
SOX10	-4.15	1.15	-5.40
SPARC	-2.13	1.03	-2.05
SPP1	-2.23	-1.51	-1.30
STAT3	-1.06	-2.29	1.32
STEAP1	-1.07	1.15	1.56
TCF3	-1.67	-1.22	1.32
TCF4	-1.67	-1.36	-1.11
TFPI2	-1.12	1.08	1.40
TGFB1	-1.51	-1.47	-2.94
TGFB2	-1.67	-1.23	1.41
TGFB3	-1.58	1.65	-1.91
TIMP1	-1.18	1.24	1.64
TMEFF1	-1.65	1.05	1.05
TMEM132A	-1.48	-3.52	-1.15
TSPAN13	-1.24	1.39	-1.25
TWIST1	-1.64	-1.08	-1.83
VCAN	-4.21	4.30	-3.69
VIM	-1.08	-1.12	1.33
VPS13A	-1.62	-1.08	-1.05
WNT11	1.56	1.86	1.63
WNT5A	-1.61	-1.53	-2.24
WNT5B	1.00	3.31	1.03
ZEB1	-1.96	-1.02	-1.18
ZEB2	-1.86	-1.14	-1.58

Batth et al

Sham castrated control	Normal	LGPIN	HGPIN	Ca
8625	—	—	—	+
8627	—	—	—	+
8628	—	—	—	+
8629	—	—	—	+
8630	—	—	+	—
5981	—	—	—	+
5982	—	—	—	+
5983	—	—	—	+
5985	—	—	—	+
5989	—	—	—	+
**Sham castrated low dose**	**Normal**	**LGPIN**	**HGPIN**	**Ca**
4897	—	—	+	—
8661	—	—	—	+
8669	—	+	—	—
8681	—	—	+	—
8683	—	—	—	+
8685	—	—	—	+
5923	Small prostate			
5926	—	—	—	+
5928	—	—	+	—
5930	—	—	+	—
**Sham castrated high dose**	**Normal**	**LGPIN**	**HGPIN**	**Ca**
5961	—	—	—	+
5969	+	—	—	—
8638	—	+	—	—
8632	—	—	+	—
8633	+	—	—	—
8636	—	—	—	+
8639	+	—	—	—
8640	—	—	—	+
8691	—	+	—	—
8700	—	—	—	+

Castrated control An.ID	Normal	LGPIN	HGPIN	Ca
8647	+	—	—	—
8648	+	—	—	—
8649	—	—	—	+
8650	+	—	—	—
5952	—	—	—	+
5953	—	+	—	+
5954	—	—	+	—
5956	—	—	—	+
5958	+	—	—	—
5960	—	—	—	+
Castrated low dose	Normal	LGPIN	HGPIN	Ca
5932	+	—	—	—
5933	—	—	—	+
5936	+	—	—	—
5938	+	—	—	—
5939	+	—	—	—
5940	—	—	—	+
8651	—	—	—	+
8652	—	—	—	+
8653	—	—	—	+
8654	—	+	—	—
Castrated high dose	Normal	LGPIN	HGPIN	Ca
8657	—	—	—	+
8659	+	—	—	—
8660	—	—	—	+
5971	—	—	+	+
5972	—	—	+	—
5973	—	—	+	—
5975	—	—	—	+
5976	—	—	—	+
5979	—	—	—	+
5980	Small prostate			

Group	*n*	Group ID	Description
1	5	S-Ctrl	Sham castrated – no drug treatment
2	5	S-Low	Sham castrated – low dosage treatment
3	5	S-High	Sham castrated – high dosage treatment
4	5	C-Ctrl	Castrated – no drug treatment
5	5	C-Low	Castrated – low dosage treatment
6	5	C-High	Castrated – high dosage treatment

Supplementary figure 1: A. Violin plot showing expression of MST1R in primary prostate tumor tissues classified by pathological stages extracted from TCGA_PRAD dataset (n = 486, PanCancer Atlas). Each dot represents a single tumor tissue.

B. Expression of MST1R, AR and KLK3 in LNCaP and MDA PCa 2b were extracted from the Cancer Cell Line Encyclopedia (CCLE) data [80]. A heatmap was created based on relative overexpression (red) and under expression (green) using the Multi-Experiment Viewer (MeV 4.9) software.

C. 2-ME_2 treatment in PC-3 cells affects multiple genes associated with EMT. A 96-well qPCR array of genes associated with EMT was screened using cDNA derived from control or 24h 2-ME_2 treated (3μM) PC-3 cells. Each column represents a single experiment. Data shows fold change in gene expression relative to β-actin. A heatmap was created based on relative overexpression (red) and under expression (green).

Supplementary figure 2: Pathological analysis of prostate tumors from individual animals from preclinical study.

Supplementary figure 3: Experimental description of metabolomic analysis from serum samples.
